# Hepatitis B virus mutation pattern rtL180M+A181C+M204V may contribute to entecavir resistance in clinical practice

**DOI:** 10.1080/22221751.2019.1584018

**Published:** 2019-03-08

**Authors:** Yan Liu, Yi Zhou, Xiaodong Li, Ming Niu, Rongjuan Chen, Jinman Shao, Lanlan Si, Dan Luo, Yayun Lin, Le Li, Kai Zhang, Xiaohe Xiao, Zhihui Xu, Min Liu, Mengji Lu, Fabien Zoulim, Dongping Xu

**Affiliations:** a Institute of Infectious Diseases, Beijing 302 Hospital, Beijing, People’s Republic of China; b Department of Infectious Diseases, The First Affiliated Hospital of Xi’an Jiaotong University, Xi’an, People’s Republic of China; c Institute of Chinese Medicine, Beijing 302 Hospital, Beijing, People’s Republic of China; d Institute of Virology, University Hospital of Essen, University of Duisburg-Essen, Essen, Germany; e Univ Lyon, Universite Claude Bernard Lyon 1, INSERM 1052, CNRS 5286, Centre Leon Berard, Centre de recherche en cancerologie de Lyon, Lyon, France; f Department of Hepatology, Groupement Hospitalier Nord, Hospices Civils de Lyon, Lyon, France

**Keywords:** Hepatitis B virus, nucleoside/nucleotide analogues, entecavir resistance, mutation, rtA181C, antiviral therapy

## Abstract

**Background and Aims:** Entecavir (ETV) resistance of hepatitis B virus (HBV) conventionally requires rt184, 202, or 250 mutations plus lamivudine-resistance mutation (rtM204V/I ± L180M). This study aimed to clarify whether rtL180M+A181C+M204V mutations may contribute to HBV ETV resistance.

**Methods:** Serum samples were collected from 22,009 patients who underwent resistance testing in Beijing 302 Hospital from 2007 to 2016. HBV reverse transcriptase (RT) gene was screened by direct sequencing and verified by clonal sequencing. Phenotypic analysis was performed for evaluating replication capacity and drug susceptibility.

**Results:** Classical ETV-resistance mutations of HBV were detected in 1252 patients who were receiving ETV therapy. The rtA181C mutation was detected with rtL180M+M204V mutations in 18 lamivudine-experienced ETV-treated patients, and the emergence of the mutations was associated with virological breakthrough or inadequate virological response to ETV. Patient-derived representative rtA181C-containing mutants, rtL180M+A181C+M204V, rtL180M+A181C+M204V+M250V, and rtL180M+A181C+S202G+M204V, exhibited 45.7%, 25.9%, and 25.0% replication capacity and 85.6-, 356.1-, and 307.1-fold decreased susceptibility to ETV respectively compared to the wild-type strain, while the three mutants remained sensitive to tenofovir (TDF). Artificial elimination of rtA181C largely restored the rtL180M+A181C+M204V mutant’s sensitivity to ETV. Molecular modelling of viral RT binding to ETV showed that the rtL180M+A181C+M204V mutant had a less stable conformation compared to rtL180M+M204V mutant. In clinical practice, undetectable serum HBV DNA was achieved in two of five longitudinally followed rtA181C-positive patients who received switching-to TDF therapy, but not in the other three who received add-on adefovir therapy during observation.

**Conclusions:** Both clinical and experimental data support rtL180M+A181C+M204V as a novel non-classical ETV-resistance mutation pattern.

## Introduction

It has been estimated that there are 240 million people with chronic HBV infection worldwide and around 78 million of them are in China [1,2]. Chronic HBV infection may cause chronic hepatitis B that progresses to liver cirrhosis, liver failure, and hepatocellular carcinoma. Treatment for chronic hepatitis B is aimed at suppressing viral replication to the lowest possible level, thereby halting the progression of liver disease [3]. Nucleoside and nucleotide analogues (NAs) are major anti-HBV agents used in clinical practice, which target the reverse transcriptase (RT) region of HBV polymerase and efficiently inhibit HBV replication. However, a long duration of NA treatment may be associated with an increased risk of developing drug resistance [4]. Currently, five NAs are licensed in China for treating HBV infection, lamivudine (LAM), adefovir dipivoxil (ADV), entecavir (ETV), telbivudine (LdT), and tenofovir disoproxil fumarate (TDF).

ETV is a potent antiviral, which has a high barrier to resistance and is recommended as first-line anti-HBV agent. Signature or classical ETV-resistance mutations require an HBV RT mutation at position rtT184, S202, or M250 in the presence of LAM-resistance mutations rtM204V+L180M or rtM204I ± L180M (abbreviated as LAMr) [4,5]. Although ETV resistance rarely occurred in NA-naive patients, the rates of resistance increased to 51% in LAM-refractory patients [6]. In addition to the classical ETV-resistance mutations, a novel mutation pattern, rtL180M+M204V+rtA186 T(±I163V) from an ETV-refractory patient were reported to account for ETV resistance [7]. Moreover, HBV rtL269I and rtS78 T/sC69stop mutations were respectively reported being associated with enhanced viral replication of LAMr mutants and insufficient response to ETV treatment [8,9].

In recent years, we have identified several unusual HBV RT mutations associated with LAM-, ADV-, and multidrug-resistances based on genotypic analysis of a large number of NA-treated patients in combination with phenotypic analysis [10–16]. In this study, we focused on the identification of a novel HBV mutation pattern from ETV-refractory patients, i.e. rtL180M+A181C+M204V.

## Results

### Clinically prevalent profile of classical ETV-resistance mutations

HBV ETV-resistance mutations were detected in 5.69% (1,252/22,009) of total patients, and in 20.29% (1,252/6,170) of ETV-experienced patients enrolled in the study. Among the 1252 patients, only 17 patients (1.36%) had no exposure history to other nucleotide analogues such as LAM and LdT. HBV genotypes B, C, and D (HBV/B, HBV/C, and HBV/D) were determined for 143 (11.42%), 1096 (87.54%), and 13 (1.04%) patients respectively. Specifically, rtT184, rtS202, rtM250, rtT184+S202, rtT184+M250, and rtS202+M250 substitution-based mutation types accounted for 41.29%, 35.86%, 14.38%, 7.35%, 0.72%, and 0.40% of ETV-resistance mutations, respectively. The patterns of LAMr, in the composition of ETV-resistance mutations, included rtM204V+L180M (82.91%), rtM204I ±L180M (15.25%), and rtM204V /I+L180M (1.84%) (Table 1). The nucleotide change for rtA181C and rtA181V mutations is summarized in Supplementary Table 1.

**Table 1.** Classical ETV-r mutation patterns from 1252 patients with chronic HBV infection.

ETV-r type	Mutation pattern	Number	ETV-r type	Mutation pattern	Number
rtT184^sub^ (*n* = 517)	rtT184A+M204V+L180M	97	rtM250^sub^ (*n* = 180)	rtM250V+M204V+L180M	61
	rtT184A+M204I+L180M	2		rtM250V+M204I ± L180M	3
	rtT184A+M204V/I+L180M	3		rtM250V+M204V/I+L180M	1
	rtT184L+M204V+L180M	231		rtM250L+M204V+L180M	21
	rtT184L+M204I ± L180M	6		rtM250L+M204I ± L180M	76
	rtT184L+M204V/I+L180M	3		rtM250L+M204V/I+L180M	2
	rtT184A/L+M204V/I+L180M	1		rtM250I+M204V+L180M	3
	rtT184S+M204V+L180M	46		rtM250I+M204I±L180M	11
	rtT184S+M204I±L180M	16		rtM250I+M204V/I+L180M	1
	rtT184S+M204V/I+L180M	2		rtM250V/L+M204V/I+L180M	1
	rtT184A/S+M204V+L180M	1	rtT184+S202^sub^ (n = 92)	rtT184A+S202G+M204V+L180M	20
	rtT184A/S+M204I+L180M	1		rtT184L+S202G+M204V+L180M	31
	rtT184F+M204V+L180M	6		rtT184A/L+S202G+M204V+L180M	2
	rtT184I+M204V+L180M	25		rtT184I+S202G+M204V+L180M	38
	rtT184I+M204I+L180M	5		rtT184I+S202G+M204V	1
	rtT184I+M204I	69	rtT184+M250^sub^ (*n* = 9)	rtT184A+M250V+M204V+L180M	2
	rtT184I+M204V/I+L180M	1		rtT184L+M250V+M204V+L180M	5
	rtT184I+M204V/I	2		rtT184L+M250L+M204V+L180M	2
rtS202^sub^ (*n* = 449)	rtS202G+M204V+L180M	441	rtS202+M250^sub^ (*n* = 5)	rtS202G+M250V+M204V+L180M	2
	rtS202G+M204I+L180M	2		rtS202G+M250I+M204V+L180M	1
	rtS202G+M204V/I+L180M	6		rtS202G+M250L+M204V+L180M	2

Note: ETV-r: entecavir-resistance; sub: substitution(s). All ETV-resistance mutation patterns contain LAM-resistance mutations.

### rtA181C mutation profile and clinical data

The rtA181C mutation was detected in 18 patients by direct sequence analysis, encompassing 0.08% (18/22,009) of the study population and 0.29% (18/6170) of ETV-experienced patients. All rtA181C-positive patients experienced various sequential/combined NAs therapies including ETV and had a history of LAM exposure prior to ETV treatment. The median duration for ETV therapy was 39 (7–96) months before rtA181C was detected. Coexistence of rtL180M+M204V mutations was detected in all rtA181C-positive patients. Clonal sequencing verified that rtL180M+A181C+M204V mutations truly coexisted in the same viral genomes for all 18 samples. In addition, the colocalization of rtL180M+A181C+M204V mutations and rtT184A or rtM250V mutation on the same viral genome was verified in the samples of three patients (P1, P2, P10). The representative cloned rtL180M+A181C+M204V sequences from each patient, together with other rtA181C-containing sequences, have been deposited in GenBank (accession number: MF682469-MF682490). Among the 18 patients, 15 (83.33%) were infected with HBV/C and three (16.67%) with HBV/B. The HBV genotype distribution showed no significant difference when compared with that of the rtA181C-negative patients across the studied population (HBV/B 14.0%, HBV/C 85.2%, HBV/D 0.8%). The clinical data and HBV mutation patterns, by direct and clonal sequencing, of the 18 patients are summarized in Table 2.

**Table 2.** Clinical information of the patients with HBV rtA181C mutation and detected mutation patterns.

Patient	Age	Genotype	HBeAg	HBV DNA (log_10_ IU/ml)	ALT (U/L)	Antiviral schedule (× month)	Direct sequencing	Clonal sequencing
P1	43	B	−	6.99	48	IFN12 → LAM12 → ADV48 → ETV59	rtL180M+A181C+M204V	rtL180M+A181C+M204V(18/20), rtL180M+A181C+M204V+M250V(2/20)
P2	40	C	−	2.64	23	LAM6 → NT19 → ADV16 → ETV42	rtL180M+A181C+M204V	rtL180M+A181C+M204V(8/20), rtL180M+A181V+M204V(5/20), rtL180M+A181C+M204V+M250V(7/20),
P3	50	C	−	6.30	100	LAM12 → ADV12 → ETV65	rtL180M+A181C/V+M204V	rtL180M+A181C+M204V(17/21), rtL180M+A181V+M204V(4/21)
P4	45	B	−	4.87	78	LAM40 → ADV12 → ETV30	rtL180M+A181C+M204V	rtL180M+A181C+M204V(16/20), wild-type(4/20)
P5	46	C	−	7.74	18	LAM21 → ADV50 → ETV36	rtL180M+A181C/V+M204V	rtL180M+A181C+M204V(12/20), rtL180M+A181V+M204V(5/20), wild-type(3/20)
P6	51	C	+	7.34	231	LAM12 → ADV24 → ETV72	rtL180M+A181C+M204V	rtL180M+A181C+M204V(20/20)
P7	56	C	+	4.76	29	LAM24 → ETV+ADV10	rtL180M+A181C+M204V	rtL180M+A181C+M204V(20/20)
P8	49	C	+	4.14	54	ADV24 → ADV+LAM29 → ETV10	rtL180M+A181C/V+M204V	rtL180M+A181C+M204V(14/21), rtL180M+A181V+M204V(7/21)
P9	45	C	-	4.58	56	LAM12 → ADV84 → ADV+ETV12	rtL180M+A181C+M204V	rtL180M+A181C+M204V(20/20)
P10	45	C	+	8.50	56	IFN12 → LAM12 → ADV60 → ETV38	rtL180M+A181C+M204V	rtL180M+A181C+M204V(19/20), rtL180M+A181C+T184A+M204V (1/20)
P11	49	C	+	3.75	34	ADV13 → LAM+ADV30 → ETV45	rtL180M+A181C+M204V	rtL180M+A181C+M204V(20/20)
P12	51	C	+	4.57	63	LAM14 → ADV20 → ETV23 → ETV+ADV68	rtL180M+A181C+M204V	rtL180M+A181C+M204V(18/20), rtL180M+T184A+M204V(2/20)
P13	40	B	−	1.60	31	LAM20 → LAM+ADV12 → ETV35	rtL180M+A181C+M204V	Cloning failed
P14	46	B	−	3.67	93	LAM36 → ADV14 → ETV30 → ETV+ADV13	rtL180M+A181C+M204V	rtL180M+A181C+M204V(20/20)
P15	55	C	+	3.33	42	LAM+ADV18 → ETV40	rtL180M+A181C+M204V	rtL180M+A181C+M204V(12/22), rtL180M+M204V(6/22), rtL180M+S202G+M204V(4/22)
P16	41	C	+	2.83	50	LAM24 → ADV12 → ETV60 → ADV+ETV36	rtL180M+A181C+M204V	rtL180M+A181C+M204V(16/20), wild-type(4/20)
P17	37	B	−	1.95	27	LAM20 → ADV24 → ETV30	rtL180M+A181C+M204V	rtL180M+A181C+M204V(20/20)
P18	51	C	+	2.62	25	LAM36 → LAM+ADV12 → ETV7	rtL180M+A181C+M204V	rtL180M+A181C+M204V(19/20), rtL180M+A181V+M204V(1/20)

Note: IFN: interferon-α; LAM: lamivudine; ADV: adefovir dipivoxil; ETV: entecavir; NT: not treated with antivirals.

### Clinical course of five representative cases with rtA181C substitution and HBV mutant evolution during antiviral treatment

Serial serum samples were obtained from five rtA181C-positive patients (patient 1 through patient 5 in Table 2) whose clinical information, and dynamic changes of their mutant viruses, were longitudinally analysed.

**Figure 1.**
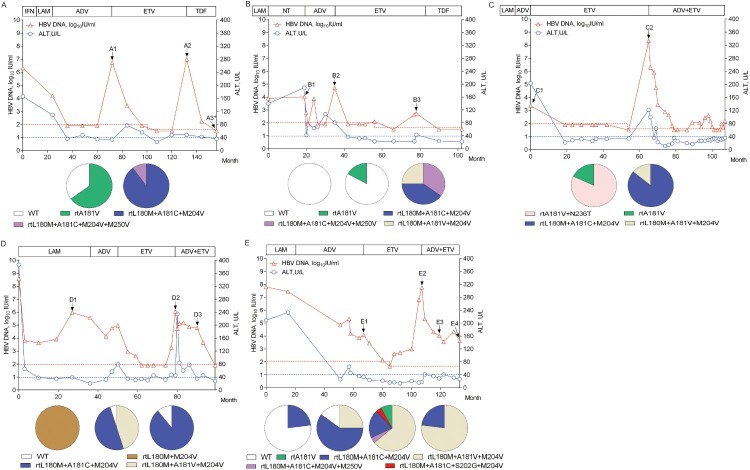
The evolution of drug-resistance HBV and clinical responses during the antiviral therapies for representative patients (*n* = 5). The dynamic changes of serum HBV DNA and alanine aminotransferase (ALT) levels are shown along with the antiviral therapies. The durations (months) of the antiviral therapies is indicated by the bars above the graph and serum samples from the patient are indicated by the sample (A−E) numbers below the graph. Two dashed lines show the lower detection limit of HBV DNA in two successive periods in clinic (100, 40 IU/mL) and normal ALT level (40 U/L). Proportions of wild-type (WT) and mutant HBV strains in the viral reverse transcriptase from each sample are depicted by a series of pie charts. IFN: interferon-α; LAM: lamivudine; ADV: adefovir dipivoxil; ETV: entecavir; and TDF: tenofovir disoproxil fumarate. NT: not treated with antivirals.

Patient 1 initially received sequential LAM and ADV monotherapies and then switching to ETV. Clonal sequencing of serum sample A2 at virological breakthrough showed that rtL180M+A181C+M204V and rtL180M+A181C+M204V+M250V mutants accounted for 90% and 10% of the tested viral clones, respectively. Subsequent TDF rescue therapy suppressed HBV DNA to an undetectable level (<40 IU/ml) with a failure of sequence analysis of sample A3 (Figure 1(A)).

Similarly, patient 2, 3, and 4 successively received LAM, ADV, and ETV monotherapies. For patient 2, clonal sequencing of serum sample B3 at virological breakthrough during ETV treatment showed that rtL180M+A181C+M204V, rtL180M+A181C+M204V+M250V, and rtL180M+A181V+M204V mutants accounted for 40%, 35%, and 25% of tested viral clones, respectively (Figure 1(B)). For patient 3, clonal sequencing of serum sample C2 at virological breakthrough during ETV treatment showed that rtL180M+A181C+M204V mutants were 86% concomitant with 14% of the rtL180M+A181V+M204V mutants (Figure 1(C)). For patient 4, clonal sequencing of serum sample D2 at virological breakthrough during ETV treatment showed that rtL180M+A181C+M204V and rtL180M+A181V+M204V mutants and wild-type strains accounting for 50%, 45%, and 5% of the tested viral clones, respectively (Figure 1(D)).

Patient 5 also initially received sequential LAM, ADV, and ETV monotherapies. Clonal sequencing of serum sample E2 at virological breakthrough during ETV treatment showed that rtL180M+A181C+M204V and rtL180M+A181V+M204V mutants and wild-type strains accounted for 60%, 25%, and 15% of the tested viral clones, respectively. Afterwards, therapy was switched to an ADV+ETV Clonal sequencing of serum samples E3 and E4 during the combination therapy showed that the rtL180M+A181V+M204V mutant predominantly emerged (64% in E3, 77% in E4) in concomitance with rtL180M+A181C+M204V mutants (20% in E3, 23% in E4), rtA181V mutants (8% in E3), rtL180M+A181C+M204V+M250V mutants (4% in E3), and rtL180M+A181C+S202G+M204V mutants (4% in E3) (Figure 1(E)).

### Phylogenetic tree analysis

**Figure 2.**
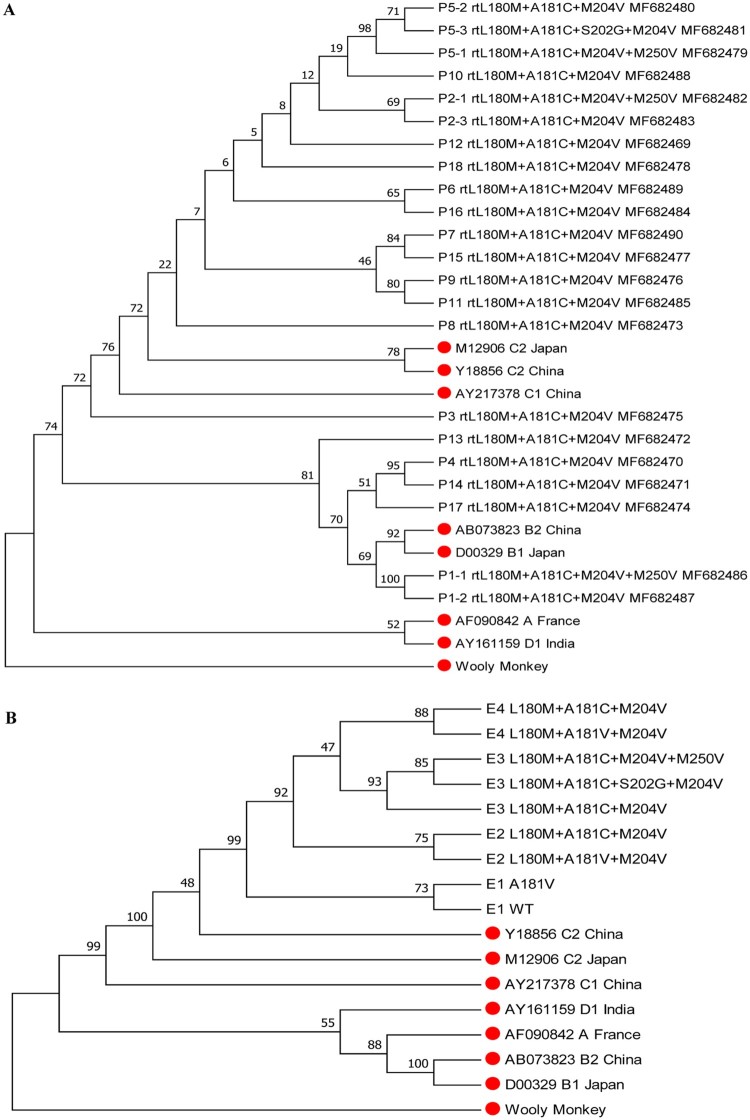
Phylogenetic tree analysis for HBV RT sequences from rtA181C-positive patients. (A) Analysis for 22 RT sequences containing rtL180M+A181C+M204V mutations from the 18 patients. P represents patient. (B) Analysis for nine RT sequences from serial serum samples of a representative patient (patient 5). The reference sequences are marked with red solid circle.

Phylogenetic tree analysis was performed for the 22 cloned HBV RT gene sequences that harboured rtA181C mutation, as well as for the nine cloned RT gene sequences from serial serum samples of patient 5. The results showed that the viral sequences from five patients (P1, P4, P13, P14, and P17) were classified as genotype B, and the sequences from the other 13 patients were classified as genotype C (Figure 2(A)). The viral sequences from samples E1 to E4 of patient 5 exhibited successive evolutionary relationship (Figure 2(B)).

### Replication capacity and drug susceptibility of rtA181C mutants

**Figure 3.**
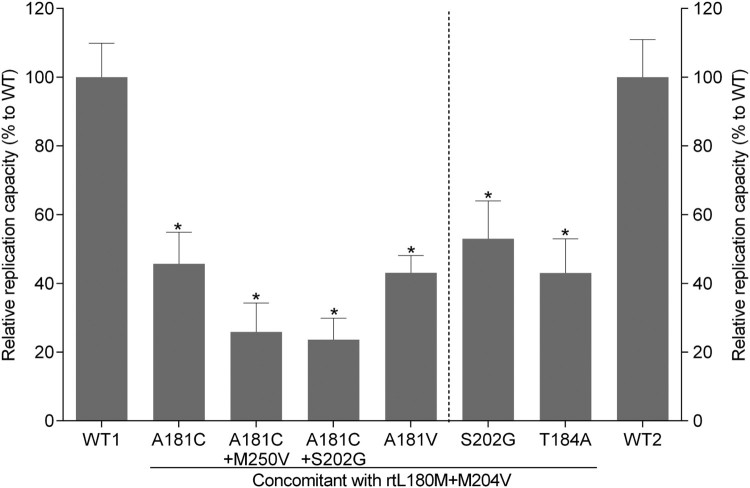
Assessment of HBV natural replication capacity. The relative replication capacities of one wild-type (WT1) and four mutant strains isolated from serial samples from a representative patient (patient 5) were analysed compared to that of the wild-type strain (100%) in the absence of drug treatment (right part). Two classical entecavir-resistance mutants (rtL180M+S202G+M204V, rtL180M+T184A+M204V) and one wild-type strain (WT2) from another entecavir-refractory patient were taken as references for the analysis (left part). Data are presented as the mean ± standard deviation. Experiments were performed at least three times independently. *P < .05.

Phenotypic analysis was performed for four mutants and one wild-type strain derived from serial serum samples of patient 5, as well as for two classical ETV-resistance mutants and one wild-type strain of genotype C HBV (GenBank accession number: GQ402156, GQ402157, and GQ402151) derived from another ETV-refractory patient [16]. In addition, a laboratory strain rtL180M+M204V(lab) which was generated by eliminating rt181C mutation of the rtL180M+A181C+M204V was taken into the phenotypic analysis of drug resistance. Compared to the wild-type strain, rtL180M+A181C+M204V and rtL180M+A181V+M204V mutants had a modest decrease in viral replication capacity (45.7% and 43.1% of the wild-type, respectively). The two classical ETV-resistance mutants also exhibited a modest decrease in viral replication (53.0% and 43.0% of the wild-type, respectively). In contrast, replication capacity decreased to a greater extent in rtL180M+A181C+M204V+M250V and rtL180M+A181C+S202G+M204V mutants (25.9% and 25.0% of the wild-type, respectively) (Figure 3). The rtL180M+A181C+M204V, rtL180M+A181C+M204V+M250V, rtL180M+A181C+M204V+S202G, and rtL180M+A181V+M204V exhibited 85.6-, 356.1-, 307.1-, and 15.0-fold decreased susceptibility to ETV, respectively. Elimination of rtA181C from the rtL180M+A181C+M204V mutant led to a restoration of the ETV-resistance level from 85.6-fold to 17.9-fold, a level similar to that of the rtL180M+A181V+M204V mutant (15.0 folds). The two classical ETV-resistance mutants exhibited 137.7- and 110.4-fold decreased susceptibility to ETV, respectively. The rtA181C-containing mutants had a 2.7 − 3.2-fold decrease in TDF susceptibility which was lower than that of the rtL180M+A181V+M204V mutant but similar with that of the two classical ETV-resistance mutants. The results are summarized in Table 3. Consistently, Southern blotting analysis verified that the rtL180M+A181C+M204V mutant had a 70.5-fold increased EC_50_ of ETV (representing a 70.5-fold ETV resistance) compared to the wild-type (Supplementary Figure 1).

**Table 3.** Drug susceptible analysis of representative HBV strains.

Viral strain	Entecavir		Tenofovir	
	EC_50_ (μmol/L)	Fold	EC_50_ (μmol/L)	Fold
Wild-type 1	0.004 ± 0.006	1.0	1.828 ± 0.926	1.0
rtL180M+A181C+M204V	0.357 ± 0.083	85.6	5.453 ± 1.004	3.0
rtL180M+A181C+M204V+M250V	1.487 ± 0.219	356.1	4.927 ± 0.971	2.7
rtL180M+A181C+S202G+M204V	1.282 ± 0.495	307.1	5.904 ± 2.062	3.2
rtL180M+A181V+M204V	0.060 ± 0.035	15.0	13.002 ± 3.584	7.1
rtL180M+M204V(lab)	0.075 ± 0.036	17.9	3.090 ± 0.928	1.7
Wild-type 2	0.003 ± 0.001	1.0	0.710 ± 0.220	1.0
rtL180M+S202G+M204V	0.413 ± 0.454	137.7	1.548 ± 0.328	2.2
rtL180M+T184A+M204V	0.331 ± 0.744	110.4	1.426 ± 0.306	2.0

Notes: EC_50_: the 50% effective concentration of drug. Fold: the EC_50_ of mutant/the EC_50_ of wild-type. Wild-type 1 and four subsequent mutant strains were derived from serial serum samples of an rtA181C-positive patient. rtL180M+M204V(lab) was a laboratory strain created by eliminating rtA181C mutation from the rtL180M+A181C+M204V mutant. Wild-type 2 and two subsequent classical entecavir-resistance mutant strains were derived from another entecavir-refractory patient.

### Molecular modelling of HBV RT binding to ETV-TP

**Figure 4.**
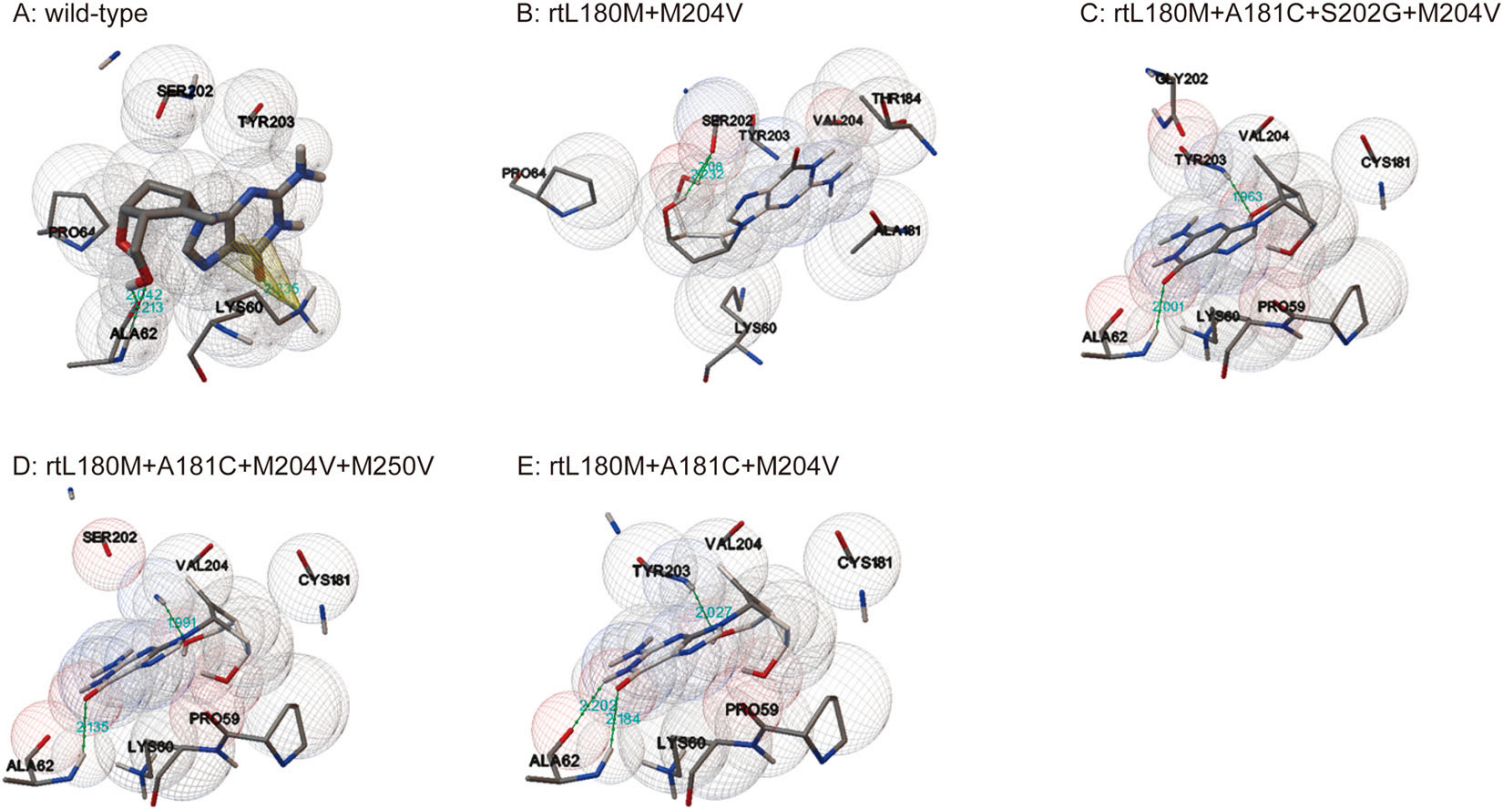
Three dimensional structures of the entecavir triphosphate-binding domains of viral reverse transcriptase (RT). The effects of ETV-resistance mutations on the binding ability of HBV RT to ETV-TP were evaluated using a homology model constructed based on the crystal structure of HIV RT. The binding domains of a wild-type and four individual mutants are presented in the order of A, B, C, D, and E. Spheres represent HBV molecular surfaces. Green dot lines represent hydrogen bonds, and yellow net represents pi-cation interaction.

The effects of ETV-resistance mutations on the binding ability of HBV RT to ETV-TP were evaluated using Autodock software. The modelling structures of the wild-type HBV RT sequence and the HBV RT mutant sequences containing rtA181C plus LAMr, each of which binds to ETV-TP, are shown in Figure 4. Consistent with the fact that the lower binding energy signifies the more stable conformation, the wild-type RT domain had the lowest binding energy ( 5.26 kcal/mol), binding to ETV-TP by three hydrogen bonds (one O–H:O hydrogen bond and two N–H:O bonds) and one pi-cation bond. The rt180M+M204V-containing mutants changed binding site and bond of RT domain to ETV-TP, and clearly increased the binding energy. Specifically, the rtL180M+A181C+M204V mutant had higher binding energy compared to the rtL180M+M204V mutant (−4.56 kcal/mol *vs.* −4.97 kcal/mol). Additional introduction of rtS202G or rtM250V into rtL180M+A181C+M204V mutation pattern resulted in one N–H:O bond reduction with further increased binding energy (Table 4).

**Table 4.** Binding energy and hydrogen bonds of viral RT to ETV-TP.

HBV strains	Binding energy (Δ*G*: kcal/mol)	N–H:O bonds	O–H:O bonds	pi-cation interactions
wild-type	−5.26	2	1	1
rtL180M+M204V	−4.97	0	2	0
rtL180M+A181C+M204V	−4.56	3	0	0
rtL180M+A181C+S202G+M204V	−4.37	2	0	0
rtL180M+A181C+M204V+M250V	−4.37	2	0	0

Note: RT: reverse transcriptase; ETV-TP: entecavir triphosphate.

The results of molecular dynamics simulation suggested that all the mutant proteins showed a lower root mean square deviation than the wild-type form, which may reduce the overall flexibility of the mutant proteins and prevent the ETV-TP from interacting with them Specifically, rtL180M+A181C+M204V mutant protein had lower values than that of wild-type and rtL180M+M204V mutant proteins (Supplementary Figure 2). In addition, the root mean square fluctuation (RMSF) graph showed that the flexibility of the mutant RT was obviously reduced, indicating that rtL180M+A181C+M204V mutation led to greater protein rigidity and therefore made the HBV RT relatively unsuitable for interacting with ETV-TP (Supplementary Figure 3).

## Discussion

HBV has the potential to evolve under environmental pressure through the selection of adaptive mutations that the most fit mutants may emerge depending on their replication capacity, and their sensitivity to antivirals and to the host immune responses [17,18]. Drug resistant mutants are usually less fit, or do not replicate as well, as the wild-type virus, but may have a survival advantage under antiviral pressure [19]. In China, nonstandard anti-HBV therapy and rescue therapy used to be common in clinical practice largely due to economic and educational reasons, and this increased the occurrence and complexity of HBV drug resistance. In this study, we identified the rtA181C mutation through sequence analysis of a large number of chronic HBV-infected patients’ samples, and managed to follow-up with five of the 18 rtA181C-positive patients. The 18 patients had no link each other. Because some patients from outside Beijing only had a short stay at follow-up and they selected to be simultaneously sampled for HBV DNA quantitation and drug-resistance testing, drug-resistance testing was performed for a few of the patients even they had very low HBV DNA levels as shown in Table 2.

An HBV resistance mutation pattern has several distinguishing characteristics, including an association with drug therapy, appearance in multiple patients exposed to the drug, an association with a rebound in viremia, and an ability to confer phenotypic resistance *in vitro* [20]. Regarding phenotypic resistance, a small decrease *in vitro* ADV susceptibility (2–9-fold increase in EC_50_) may confer clinical resistance. By contrast, *a* > 10-fold (usually >50-fold) increase in EC_50_ for ETV and TDF, and *a* > 500-fold increase in EC_50_ for LAM, are needed for clinical resistance [21,22]. The rtL180M+A181C+M204V mutation pattern reported herein meets these conditions for ETV resistance: it was only detected in the patients who were receiving ETV and had experienced LAM treatment, it emerged in multiple patients with virological breakthrough or inadequate virological response against ETV monotherapy, and it conferred *a* > 50-fold increased EC_50_ for ETV. In addition, artificial elimination of rtA181C obviously restored its sensitivity to ETV, verifying the critical role of rtA181C mutation in ETV-resistance contribution.

Drug susceptibility and replication capacity are two major factors influencing the fitness of a mutant virus under drug pressure [23]. The LAMr rtL180M+M204V had decreased susceptibility to ETV but the decrease was not sufficient to cause clinical ETV resistance. Resistance requires an additional mutation at rtT184 (A, F, I, L, M, S), rtS202 (A, C, G), or rtM250 (I, L, V) [24]. On the other hand, a rtT184, rtS202, or rtM250 mutation alone has minimal effect on susceptibility to ETV, but susceptibility to ETV is decreased by 10–250 folds with LAMr, and by >500-fold when two or more of the mutants are combined with LAMr [21]. Walsh et al. [25] identified ETV-resistance features in the mutants rtL180M+M204V (LAMr), LAMr+rtT184L, LAMr+rtS202G, LAMr+rtM250V, and LAMr+rtT184G+rtS202I, showing they had 27, 246, 402, 1028, and >1333-fold respective decreases in drug susceptibility when compared to the wild-type strain. In this study, we identified that the LAMr+rtA181C (rtL180M+A181C+M204V) mutant had 85.6-fold decreased susceptibility to ETV *in vitro*, which was lower than the decrease values of two classical ETV-resistance mutants simultaneously identified in the study (137.7 and 110.4 folds, Table 3), as well as reported values above. This might partly account for the infrequency of LAMr+rtA181C-causative ETV resistance in clinic. In addition, we identified two novel ETV-resistance mutants, rtL180M+A181C+M204V+M250V and rtL180M+A181C+S202G+M204V. The two mutants successively emerged during ADV+ETV treatment (Figure 1(E)), and the rtL180M+A181C+M204V+M250V mutant also emerged in patient 1 and patient 2 during ETV treatment. They had a higher ETV resistance but much lower replication capacity compared to the rtL180M+A181C+M204V mutant that emerged previously. These data suggest that the rtM250V or rtS202G mutation contributed to the rtL180M+A181C+M204V template to adapt to selective drug pressure, while they had no persistent advantage for replication competency to overcome the continuous pressure of ADV+ETV.

Overall, the occurrence frequency of rtL180M+A181C+M204V in NAs-treated patients was rather low, and none of ETV naïve treated patients were detected with rtL180M+A181C+M204V. It has been suggested rt181 mutation (rtA181 T/V) is involved in a shared pathway for resistance of several NAs [26]. Most rtA181 T mutation only needed one-nucleotide change as we previously analysed [27], and so did for rtA181V mutation (Supplementary Table 1). In contrast, rtA181C mutation requires a two-nucleotide change (GCT to TGT), which would increase the genetic barrier of the mutation and largely explain its low clinical incidence. From the view of resistance history, the five representative rtA181C-positive patients all had rtA181V and/or rtA181V-containing mutations (as shown in Figure 1). Therefore, rtC181 (TGT) mutation might be more likely derived from V181 (GTT) rather than A181 (GCT). The successive resistance history to LAM and ADV was likely a favourite factor for the development of the rtL180M+A181C+M204V mutation, which could contribute to the rare occurrence of rtA181C as an ETV resistance mutation.

HBV genotype might be relevant in the evolution and development of drug resistance [28]. In North China, HBV/C is dominant while HBV/B is subdominant. We have recently reported that HBV/C and HBV/B-infected patients had a similar rate of ETV-resistance mutations, but different ETV-resistance mutation patterns [29]. In this study, the occurrence of an rtA181C mutation was independent of HBV genotypes. The rtL180M+A181C+M204V mutant sequences were not found being documented previously in GenBank. Unlike rtA181 T mutation which may cause sW172stop and non-stop (sW172S, sW172L) mutations and the stop mutation will delete an HLA-A2-restricted s172-180 (env335-343) epitope of cytotoxic T lymphocytes (CTL) [27] the rtA181C mutation only caused sW172C mutation in this study without deleting the CTL epitope.

Switching to TDF is currently the preferred rescue therapy for ETV resistance [1]. As TDF was not licensed for treating HBV infection until 2014 in China, ADV+ETV combination was recommended as an alternative, and a preferential rescue therapy, for ETV resistance in successively issued guidelines [30,31]. In the five ETV-resistance patients with rtL180M+A181C+M204V presented in this study, two received TDF and the other three received ADV+ETV. Comparatively, TDF looked more efficacious than ADV+ETV. Phenotypic analysis verified that three rtA181C-containing mutants only had a 2.7−3.2-fold decrease in TDF susceptibility, similar with the susceptibility of the two classical ETV-resistance mutants (2.0−2.2-fold decrease) in our study and rtL180M+S202G+M204V mutant (2.5-fold decrease) reported by other investigators [32].

The modelling of viral RT indicated that the introduction of rtA181C mutation into LAMr mutant changed hydrogen bonds from O–H:O form to relatively less stable N–H:O form, leading to the decrease of the binding affinity of HBV RT to ETV-TP. Addition of classical ETV-resistance mutation rtS202G or rtM250V into LAMr+rtA181C mutant reduced N–H:O hydrogen bonds from three to two, leading to further decrease of the binding affinity of HBV RT to ETV-TP. The results of molecular dynamics simulation also indicated LAMr+rtA181C together reduced the overall flexibility of the mutant proteins and prevented the ETV-TP from interacting with them. These modelling results reinforced that rtL180M+A181C+M204V to be a novel ETV-resistance mutation pattern analysis and supplied likelihood resistance mechanisms for the mutation pattern.

In conclusion, this study is the first to demonstrate that rtL180M+A181C+M204V is a non-classical ETV-resistance mutation pattern and identified two other rtA181C-containing ETV-resistance mutation patterns, rtL180M+A181C+S202G+M204V and rtL180M+A181C+M204V+M250V. These rtA181C mutants remained sensitive to TDF treatment. This study provides new insights into HBV drug resistance, with clinical implications for resistance management.

## Materials and methods

### Patients and samples

A total of 22,009 chronic HBV-infected patients who received resistance testing (direct sequencing) in Beijing 302 Hospital from 2007 to 2016 were enrolled, and these patients had all received NAs treatment (including 6170 ETV-experienced patients) as described previously [28]. The patients were from different regions of China, including around 87% from North China and 13% from South China. The illness categories of chronic HBV infection included chronic hepatitis B, HBV-related liver cirrhosis, and hepatocellular carcinoma. The standards for diagnosing these illnesses categories were based on the Guideline of Prevention and Treatment for Chronic Hepatitis B issued by the Chinese Society of Infectious Diseases and Parasitology, Chinese Society of Hepatology [33]. Patients who were co-infected with other hepatitis viruses, or HIV, were excluded. These patients were from the Database of Beijing 302 Hospital, and all provided their informed consent for the use of their samples for research before enrolment in the Database of Beijing 302 Hospital. The study was approved by the Ethics Committee of Beijing 302 Hospital.

### Serological markers, quantitation of HBV DNA, and sequencing of HBV RT gene

Biochemical and serological markers, and HBV DNA level, of the patients were routinely detected in the Central Clinical Laboratory of Beijing 302 Hospital. HBV DNA level was determined using a real-time quantitative PCR kit (Fosun Pharmaceutical Co., Ltd., Shanghai, China) with a lower limit of detection (LLOD) of 100 IU/ml before April 2012 and 40 IU/ml afterwards. If sufficient amount of serum was available, HBV DNA of interesting samples below the LLOD was further quantitated using AmpliPrep/COBAS TaqMan (Roche Diagnostics) with an LLOD of 20 IU/ml as described previously [34]. For HBV resistance testing, HBV DNA was extracted from patient serum with DNA out (Tianenze, Beijing, China) and the HBV RT gene was amplified by nested PCR with an LLOD of 10 IU/ml [27]. The PCR products were directly sequenced for all samples. Clonal sequencing was performed (≥20 clones/sample at each time point) if necessary. Sequencing was performed by a professional company using an ABI 3730xl DNA Analyzer (Applied Biosystems, Foster City, CA, USA).

### HBV genotypic resistance testing, clonal sequencing, and genotype classification

HBV genotypic resistance testing was performed using direct sequencing of a 1225-bp-long viral gene fragment [nucleotide (nt) 54-1278] that covers the full-length RT region (nt 130-1161) and overlapping S region (nt 155-835). Clonal sequencing was performed using the TA cloning strategy, and drug-resistance mutations were analysed as described previously [15,16]. HBV genotype assignment was based on a phylogenetic analysis of the RT/S-gene sequence. Phylogenetic trees were constructed using neighbor-joining analysis with bootstrap test confirmation performed on 1000 resampling standard reference sequences acquired from the online hepatitis virus database of the National Institutes of Health.

### Construction of recombinant vectors containing 1.1 mer HBV genome

Viral strains from patient 5 were selected for phenotypic analysis. Considering the natural evolutionary features of the mutants during long-term NA treatments, we used clinically derived viral strains. Because in the serial samples of patients 5, rtL180M+M204V strain was not detected, and also because the need of confirmation of rtA181C’ contribution in ETV-resistance by reverse genetic method, site-directed mutagenesis was performed to generate a laboratory rtL180M+M204V strain using clinically derived rtL180M+A181C+M204V strain as the template. To increase reliability, two ETV-resistance mutants and one wild-type from another patient were examined simultaneously. pTriEx-mod-1.1 vector was used, which contains 1.1 mer genotype C HBV genome and was developed for HBV phenotypic analysis [35,36]. Recombinant vectors that harboured a patient-derived rtL180M+A181C+M204V, rtL180M+A181C+M204V+S202G, rtL180M+A181C+M204V+M250V mutant and wild-type RT genes, as well as another patient-derived rtL180M+S202G+M204V, rtL180M+T184A+M204V, and wild-type RT genes, were constructed for phenotypic analysis based on the pTriEx-mod-1.1 vector.

### Assessment of viral replication capacity and drug susceptibility

The phenotypic analysis was performed as previously described [12,13]. Briefly, recombinant vectors that harboured either mutant or wild-type HBV genome were transiently transfected into HepG2 cells and cultured in the presence or absence of serially diluted NAs. The transfection was mediated by X-tremeGENE HP DNA transfection (Roche, Mannheim, Germany) and transfection efficiency was normalized using the β-galactosidase reporter plasmid (Promega, Madison, WI, USA). Five hours post-transfection, new medium containing serially diluted NAs was supplemented every other day. Four days after cultivation, cells were harvested and lysed. Viral core particles were immunoprecipitated using anti-HBc/protein A+G. HBV replicative intermediates in core particles were released and quantitated by real-time PCR (Chinese patent ZL 2013103921225). Relative replication capacity of a mutant *vs.* wild-type strain was determined in the absence of NAs. Drug susceptibility was determined by comparing the 50% effective concentration of the drug (EC_50_) in a mutant *vs.* wild-type sample. In the comparison analysis, wild-type and mutant strains were derived from the same patient to avoid potential individual differences. The experiments were performed at least three times independently. In addition to the PCR-based assay, a Southern blotting-based assay was performed to determine viral replication capacity in the laboratory of the Institute of Virology, University Hospital of Essen, according to a previously described method [37].

### Site-directed mutagenesis

A QuikChange Lightning site-directed mutagenesis kit (Stratagene, La Jolla, CA, USA) was employed according to manufacturer’s instructions to eliminate rtA181C mutation on the rtL180M+A181C+M204V mutant. Briefly, the mutagenic primers anneal to the same sequence on opposite strands of the plasmids that were designed individually, and PCR was performed to synthesize the reverse mutant strand. The primer (sense) was 5′-CCGTTTCTCATGGCTCAGTTTACTAG-3′. The restriction enzyme *Dpn*I was used to digest parental methylated and hemimethylated DNA. The reversely mutated sequence was transformed into XL-10 competent cells for nick repair. The laboratory-modified gene was linked with the pTriEx-mod-1.1 vector as previously mentioned.

### Molecular modelling of HBV RT binding to ETV triphosphate (ETV-TP)

The modelling structure of the HBV RT was constructed by SWISS-MODEL (https://www.swissmodel.expasy.org) based on the crystal structure of the HIV RT [Protein Data Bank (PDB) accession number; 1RTD]. This HBV RT modelling structure was used as a wild-type to build the structures of the HBV NA-resistant mutants. Autodock software (version 4.2.6, molecular graphics laboratory) was used to simulate the docking process and evaluate the binding energy of HBV RT and ETV-TP. Autodock is a component of the MGLTools that is freely available for academic use. More detailed information about Autodock could be found at the following website: http://mgltools.scripps.edu/.

To further understand the conformational change of the rtA181C mutant protein, a 2000-picosecond molecular dynamics simulation was performed by NAMD (version 2.13) with a cubic box water model [38]. The root mean square deviation and RMSF were calculated and analysed using Visual Molecular Dynamics (Version 1.93) [39].

### Statistical analysis

Data were presented as the mean ± standard deviation, or the median (range). Differences between variables were examined by Student’s *t*-test. Statistical analysis was carried out in the Statistical Program for Social Sciences (SPSS 18.0 for Windows; SPSS Inc., Chicago, IL, USA). A *P*-value of <0.05 (two-tailed) was considered statistically significant.

## Supplementary Material

Supplemental Material

Supplemental Material
