# Supplementary material for: Hepatitis B virus mutation pattern rtL180M+A181C+M204V may contribute to entecavir resistance in clinical practice
Source: Emerg Microbes Infect. 2019 Mar 8;8(1):354–65. doi: 10.1080/22221751.2019.1584018 (PMC6455135; doi:10.1080/22221751.2019.1584018)
Supplement: Supplemental Material [file TEMI_A_1584018_SM5139.docx]

**Supplementary Table 1.** Analysis of the nucleotide replacements for HBV rtA181V and rtA181C mutations in 22,009 patients’ samples

| Amino acid | Nucleotide | Genotype B | | Genotype C | |
| --- | --- | --- | --- | --- | --- |
|  |  | cases | percentage | cases | percentage |
| rtA181 | GCA | 0 | 0.00% | 3 | 0.00% |
|  | GCT | 3281 | 95.30% | 16559 | 89.20% |
|  | GCC | 3 | 0.10% | 17 | 0.10% |
| rtV181 | GTC | 0 | 0.00% | 3 | 0.00% |
|  | GTT | 61 | 1.80% | 1119 | 6.00% |
|  | GTG | 0 | 0.00% | 2 | 0.00% |
| rtC181 | TGT | 47 | 1.40% | 32 | 0.20% |
| Total |  | 3443 | 98.50% | 18566 | 95.50% |
