# Supplementary figures and images for: Hepatitis B virus mutation pattern rtL180M+A181C+M204V may contribute to entecavir resistance in clinical practice
Source: Emerg Microbes Infect. 2019 Mar 8;8(1):354–65. doi: 10.1080/22221751.2019.1584018 (PMC6455135; doi:10.1080/22221751.2019.1584018)

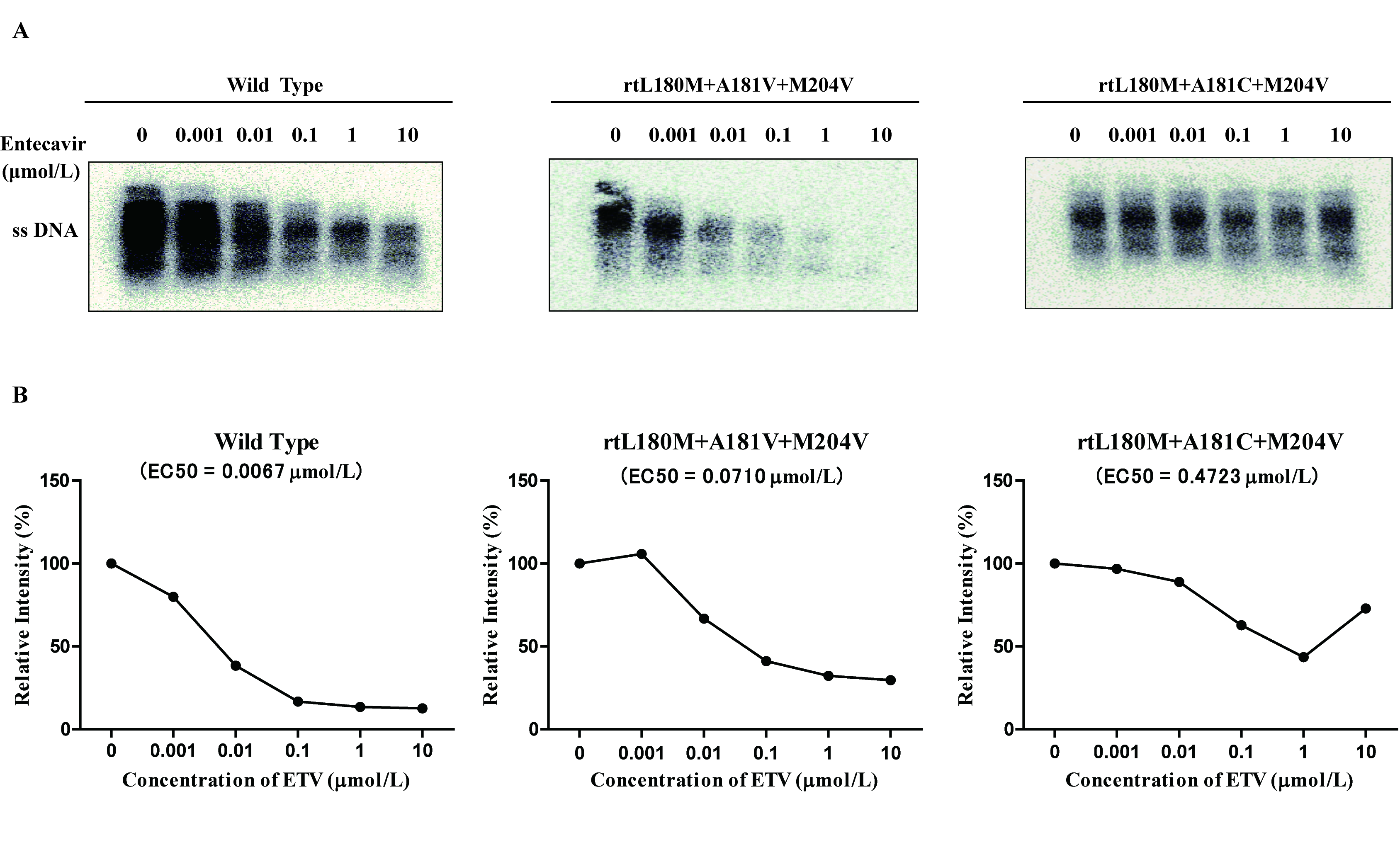

Supplement: Supplemental Material [file TEMI_A_1584018_SM4204.zip › Supplementary Material/2019-2-13 paper files to EMI-clean/2019-2-13 Supplementary Figure 1.tif]

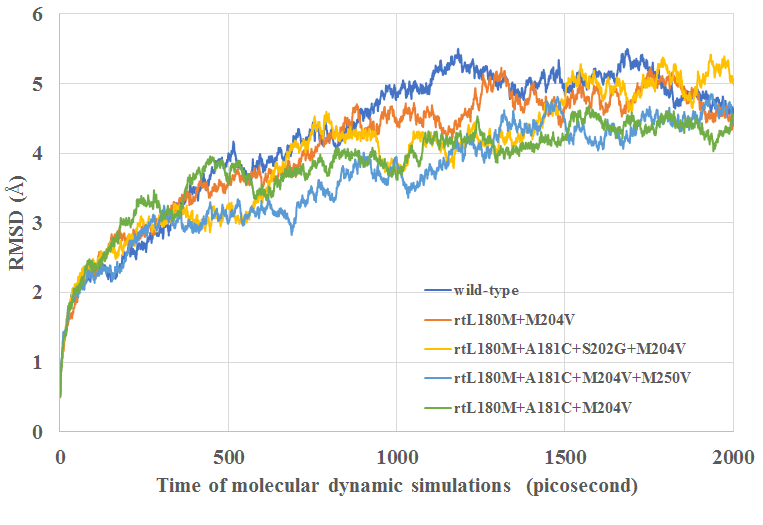

Supplement: Supplemental Material [file TEMI_A_1584018_SM4204.zip › Supplementary Material/2019-2-13 paper files to EMI-clean/2019-2-13 Supplementary Figure 2.tif]

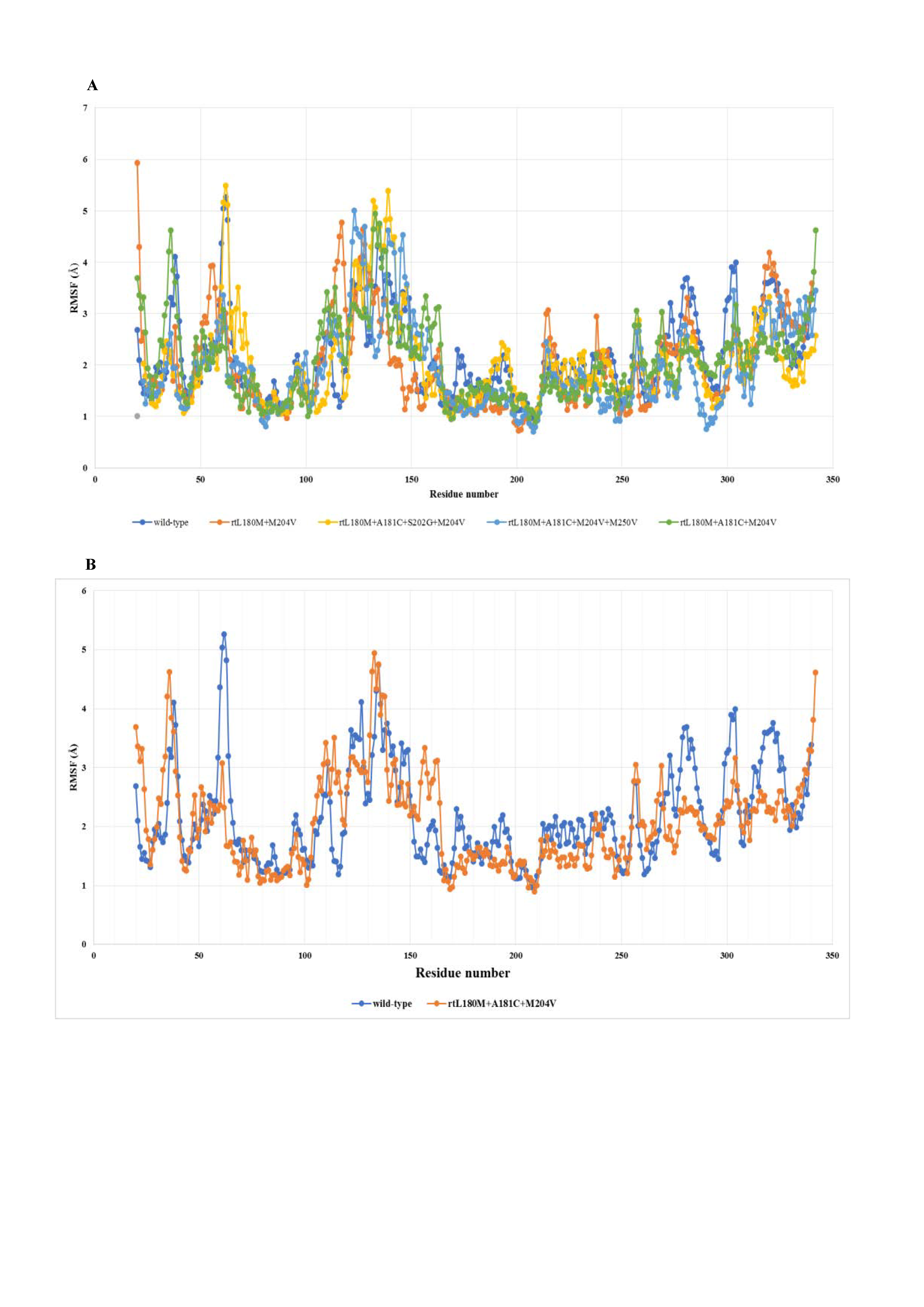

Supplement: Supplemental Material [file TEMI_A_1584018_SM4204.zip › Supplementary Material/2019-2-13 paper files to EMI-clean/2019-2-13 Supplementary Figure 3.tiff]
